# Predicting Knee Joint Kinematics from Wearable Sensor Data in People with Knee Osteoarthritis and Clinical Considerations for Future Machine Learning Models

**DOI:** 10.3390/s22020446

**Published:** 2022-01-07

**Authors:** Jay-Shian Tan, Sawitchaya Tippaya, Tara Binnie, Paul Davey, Kathryn Napier, J. P. Caneiro, Peter Kent, Anne Smith, Peter O’Sullivan, Amity Campbell

**Affiliations:** 1 School of Allied Health, Faculty of Health Sciences, Curtin University, Perth, WA 6845, Australia; Jay-Shian.Tan@curtin.edu.au (J.-S.T.); Tara.Binnie@curtin.edu.au (T.B.); P.Davey@curtin.edu.au (P.D.); JP.Caneiro@curtin.edu.au (J.P.C.); Peter.Kent@curtin.edu.au (P.K.); Anne.Smith@exchange.curtin.edu.au (A.S.); P.OSullivan@curtin.edu.au (P.O.); 2 Curtin Institute for Computation, Curtin University, Perth, WA 6845, Australia; Sawitchaya.Tippaya@curtin.edu.au (S.T.); Kathryn.Napier@curtin.edu.au (K.N.)

**Keywords:** knee osteoarthritis, machine learning, kinematics, inertial measurement unit, biomechanical analysis

## Abstract

Deep learning models developed to predict knee joint kinematics are usually trained on inertial measurement unit (IMU) data from healthy people and only for the activity of walking. Yet, people with knee osteoarthritis have difficulties with other activities and there are a lack of studies using IMU training data from this population. Our objective was to conduct a proof-of-concept study to determine the feasibility of using IMU training data from people with knee osteoarthritis performing multiple clinically important activities to predict knee joint sagittal plane kinematics using a deep learning approach. We trained a bidirectional long short-term memory model on IMU data from 17 participants with knee osteoarthritis to estimate knee joint flexion kinematics for phases of walking, transitioning to and from a chair, and negotiating stairs. We tested two models, a double-leg model (four IMUs) and a single-leg model (two IMUs). The single-leg model demonstrated less prediction error compared to the double-leg model. Across the different activity phases, RMSE (SD) ranged from 7.04° (2.6) to 11.78° (6.04), MAE (SD) from 5.99° (2.34) to 10.37° (5.44), and Pearson’s R from 0.85 to 0.99 using leave-one-subject-out cross-validation. This study demonstrates the feasibility of using IMU training data from people who have knee osteoarthritis for the prediction of kinematics for multiple clinically relevant activities.

## 1. Introduction

People who have knee osteoarthritis commonly report pain and physical limitation performing functional activities such as walking, transitioning from a chair and negotiating stairs [1]. During these activities they also use less sagittal plane range of movement (knee flexion) during particular phases of activities (e.g., stance phase of walking) compared to people who do not have osteoarthritis [2,3,4,5]. Clinicians are interested in the relationship between specific kinematic measures and clinical outcomes in people with knee osteoarthritis [6]. For example, a person may have difficulty descending stairs because they do not use available knee flexion movement during the stance phase. Interventions such as exercise [7] and total knee replacement [8] have demonstrated the ability to improve knee flexion angle during walking in people who have knee osteoarthritis. Clinical guidelines recommend that the performance of painful and limited activities are monitored over the course of treatment [9]. However, there are currently several limitations to clinicians being able to accurately quantify sagittal plane knee range of movement during functional activities in both clinical and free-living environments (e.g., patient’s home or work, or during recreation). 

Clinicians are unable to routinely access gold standard optoelectronic motion analysis systems (e.g., Vicon) due to cost and space requirements. Smartphone camera-based technology is more accessible to clinicians and has demonstrated validity and reliability for measuring sagittal plane knee angles [10]. Both optoelectronic and smartphone camera-based systems require the patient to be observed within a fixed volume to record useful clinical information, precluding their use in a free-living environment. Inertial measurement units (IMUs) are a wearable sensor technology that is emerging as an alternative for biomechanical analysis, allowing a patient to move freely in clinical and free-living environments. Multiple scoping reviews have described the potential role of IMUs for the assessment of people with knee osteoarthritis [11,12] and following knee replacement surgery [13]. These reviews highlight the need for further investigation of IMU systems that can be used for monitoring biomechanics of patients in free-living conditions. 

There is a substantial volume of research validating IMUs for estimating kinematics in laboratory environments [14,15,16], although two barriers exist for widespread clinical adoption. In uncontrolled environments such as in a clinic or in free-living conditions, the presence of metallic equipment (e.g., chairs or railings) and devices such as mobile phones and computers can interfere with the magnetometer data which can affect the reliability of fusion algorithm estimates [17,18], making the data unusable [17]. Although some fusion methods have been described which use only accelerometers and gyroscopes, they require IMU calibration prior to each use [19]. To overcome the magnetometer problem and calibration requirements, machine learning (a form of artificial intelligence) approaches have been used to predict kinematics (e.g., knee joint flexion angle) from only the raw accelerometer and gyroscope data [20,21]. Although traditional machine learning requires the researcher to identify important features from the IMU data to train the model, a more contemporary approach is to use deep learning (a subfield of machine learning) that automatically detects features, minimising programming requirements [22,23,24,25,26]. 

There are a small number of studies where deep learning models have been trained to predict knee joint angular kinematics for walking from IMU training data collected mostly from healthy people [22,23,24,25,26,27]. However, people with knee osteoarthritis experience significant difficulty with functional activities other than walking, such as negotiating stairs and transitioning to and from a chair. There is only one reported study using IMU data collected from participants who have knee osteoarthritis to train a deep learning model to predict sagittal plane knee kinematics, which was only for the activity of walking [26]. No study has yet developed a deep learning model to predict knee joint kinematics for multiple, clinically important activities using IMU data collected from people with knee osteoarthritis.

The aim of this study was to demonstrate a proof-of-concept for the feasibility of using IMU training data collected from people who have knee osteoarthritis performing three clinically relevant functional activities: walking, negotiating stairs, and transitioning to/from a chair, to train a deep learning model to predict knee joint flexion angles. The second aim was to determine if a single-leg model (two sensors on one leg) or double-leg model (two sensors on both legs) was more accurate. 

## 2. Materials and Methods

### 2.1. Study Design

This study was a retrospective, prognostic study using continuous IMU data, collected from people with knee osteoarthritis who performed multiple clinically relevant activities, to predict knee joint sagittal plane kinematics. 

### 2.2. Participants

Participants in this study were part of a broader investigation into the use of IMUs in people with knee osteoarthritis [14,28]. Seventeen participants with knee osteoarthritis were recruited from local physiotherapists, GP practices and local community centres. This number of participants mirrors other studies [21,24,29,30] and was thought to be sufficient to test the feasibility of this proof-of-concept study. Participants were included if they met the clinical diagnostic criteria for knee osteoarthritis [31], had ≥3 months of pain, ≥4/10 pain on most days, and moderate activity limitation (single item on the Function, Daily Living sub-scale of the Knee injury and Osteoarthritis Outcome Score) [32]. To minimise the effects of soft tissue artefact during motion capture that can introduce ‘noise’ into the data, we excluded participants with a body mass index (BMI) > 35 kg/m^2^ and those who had a BMI >30 kg/m^2^ with a waist-to-hip ratio (WHR) of ≤0.85 for women and ≤0.95 for men (those with greater soft tissue around the lower limbs). Participants were excluded if they had previous lower limb arthroplasty or mobility impairments due to other medical conditions (e.g., cognitive impairment, recent trauma or neurological disorders). The study was approved by the Human Research Ethics Committee of Curtin University (HRE2017-0738). 

### 2.3. Data Collection

**Figure 1:**
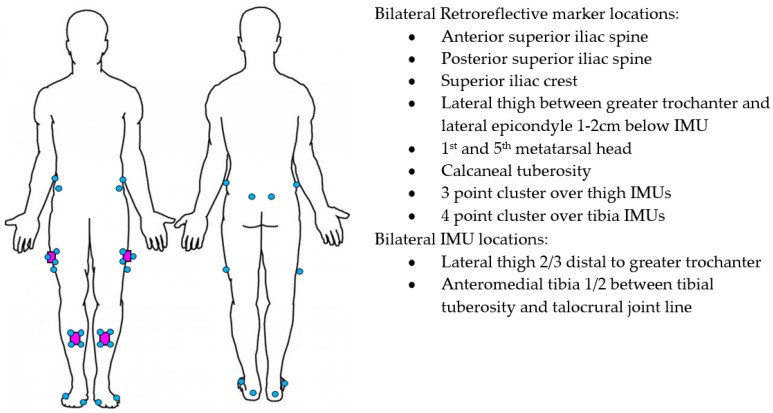
IMU (purple) and Vicon marker (blue) placement.

Participants were initially screened for eligibility over the phone and subsequently attended a university motion analysis laboratory. After providing written informed consent, height and weight data were collected. IMUs and retroreflective markers were placed on the participants in a standardised manner by an experienced musculoskeletal physiotherapist in the locations described in Figure 1. Participants performed 5 repetitions of knee flexion/extension as a warm-up on each knee. A standardised battery of functional activities was then performed that included 4 trials of stand-to-sit, 4 trials of sit-to-stand, 3 trials of 3-stair ascent, 3 trials of 3-stair descent, and 3 trials of a 5-metre self-paced walk. Participants rested for 30 s between trials and 60 s between activities. IMUs were removed after completion of the battery of functional activities and raw data were offloaded. 

### 2.4. Instrumentation

Four IMUs (v6 research sensors, DorsaVi, Melbourne, Australia) sampling at 100 Hz (accelerometer 8G, gyroscope 2000 degrees/second) were attached to the lower limbs with double-sided hypoallergenic tape. The IMUs’ dimensions were 4.8 × 2.9 × 1 centimetres and they weighed 17 g. Three-dimensional motion analysis was recorded with an 18 camera Vicon (Oxford Metrics Inc., Oxford, UK) sampling at 250 Hz. The relatively small reconstruction errors of <1 mm have resulted in the Vicon being considered the gold standard motion analysis system [33,34]. Twenty-eight retroreflective markers were placed on the participant’s pelvis and lower limbs using a cluster-based approach in alignment with International Society of Biomechanics recommendations [35]. For this purpose, marker clusters were affixed to the IMUs (Figure 1) and anatomical markers were placed at the locations outlined in Figure 1. Additional markers were applied to relevant joint centres for a static calibration trial, then removed. This approach has previously been described in detail [36]. The sensor system was synchronised with the Vicon prior to being attached to the participant. IMUs in the same orientation were placed in a wooden box with retroreflective markers attached to the outside. The box was then rotated >90° ten times and recorded as a single trial in Vicon Nexus software (Oxford Metrics Inc., Oxford, UK) to facilitate subsequent time-synchronisation of the IMU and Vicon systems. 

### 2.5. Data Preparation

Vicon trials were reconstructed and modelled using Vicon Nexus software. Gaps in trajectories were noted through visual inspection. Cubic spline interpolation was used to fill gaps of ≤20 frames (0.08 s), and if gaps were larger than this they were discarded. Kinematic trajectories were then filtered using a low-pass Butterworth filter with a 6 Hz cut-off frequency as determined by residual analysis. Vicon data were down-sampled from 250 to 100 Hz to allow time synchronisation with the IMU sensors.

We used the raw triaxial accelerometer and gyroscope data from 4 IMUs that were output as individual timestamped files using the IMU proprietary software (MDMv6 Manager v6.883, DorsaVi). Reconstructed Vicon data and the filtered raw orientation data from each IMU were time synchronised by the use of normalised cross-correlation using a customised LabVIEW program (National Instruments, Austin, TX, USA). The event markers were automatically detected by the LabVIEW program. Events for phases of walking and stair trials were heel contact and toe-off for swing and stance phases. Sit-to-stand and stand-to-sit events were anterior and posterior movement of the pelvis. Start and end times from the raw IMU data were exported for each phase of activity for the raw IMU and reconstructed Vicon data, which were used as inputs into the model. All trials were visually inspected to validate the automated synchronisation and event markers.

We input the affected leg, side of interest, activity, direction of stair climbing and phase of activity as categorical variables into the model. In this study, we also investigate the interdependency between both legs by training the model with two different structures of input data: double-leg, and single-leg. The double-leg model consisted of 38 input variables (24 accelerometer/gyroscope from 4 IMUs and 14 categorical variables), whereas the single-leg model included 27 input variables (12 accelerometer/gyroscope from 2 IMUs, side, 14 categorical variables). 

### 2.6. Deep Learning Model Development

The target prediction variable was the knee flexion joint angle at each time step obtained from the Vicon motion capture for multiple activities from the raw IMU accelerometer and gyroscope data. 

One deep learning approach known as long short-term memory (LSTM) is suitable to handle discrepancies between steps in time-series data, where each trial differs in length [37]. LSTM also requires less pre-processing compared to other deep learning approaches, such as convolutional neural networks (CNNs), and is more suitable for real-time applications [38]. Recently a N-layer feed forward neural network (FFNN) demonstrated superior results for kinematic prediction of the lower limb compared to a recurrent neural network known as LSTM [23]. FFNN generally uses all the data points to make the prediction, whereas LSTM only uses past data points, resulting in its reduced accuracy for the first few data points. We chose to use a further evolution of LSTM and FFNN known as bidirectional LSTM (BiLSTM), which has both recurrent and feedforward characteristics because it transverses the input data twice, using both past and future data for predictions [26] to improve accuracy compared to LSTM [39]. BiLSTM has been successfully implemented for predicting knee joint kinematics during walking for people who have knee osteoarthritis or previous knee replacement [26].

### 2.7. Model

A previous study using IMU data to train a deep learning prediction model reported that the number of IMUs can affect kinematic prediction error [40]. To explore the effect of using additional IMUs, we developed two models: double-leg and single-leg. The double-leg model uses data from 4 IMUs from both legs as input to predict the knee joint angle of the leg of interest, whereas the single-leg model uses data from 2 IMUs from the leg of interest as input. 

**Figure 2:**
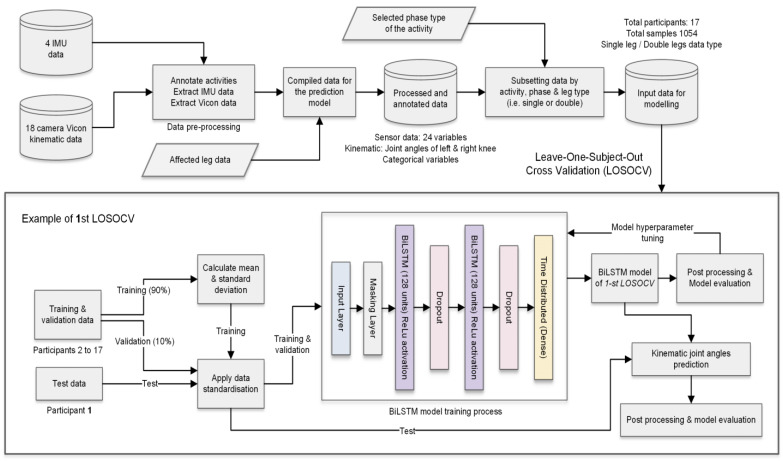
Data preparation and model architecture of the proposed BiLSTM kinematic prediction models.

The model architecture was based on a stacked BiLSTM model. As BiLSTM requires input data for each sequence to have the same length, sequences were padded to the maximum sequence length of the activity phase. A single masking layer was used as the first layer to ignore all padded values. Then, two separate BiLSTM hidden layers with 128 units and a rectified linear unit activation function extracted the features from the sequences. BiLSTM was set to output a value for each time step in the input data, resulting in returning the sequence. A dropout layer was added after each BiLSTM hidden layer to randomly drop some units together with their connections from the network to reduce overfitting. The dropout rate was set to 0.2 and learning rate 0.0001. Finally, a separate fully connected time distributed output layer with linear activation was used to return the estimated joint angle (one for a single-leg input type and two for the double-leg input type). The proposed BiLSTM kinematic prediction model architecture is illustrated in Figure 2. 

The model is trained using the adaptive momentum (Adam) optimisation algorithm [41]. The final model parameters were selected after the hyperparameter tuning process assessing the loss function and model metrics. In this study, we retained the same hyperparameters across all model training processes to investigate the influence of input data variation to the prediction model. The data processing, machine model and experimental results were developed and implemented using Python 3 with the libraries Pandas, Numpy, Scipy, Scikit learn, Keras and Tensorflow. 

### 2.8. Validation and Data Standardisation

As a clinician needs to know the average accuracy of a predictive model for each new patient, we used a leave-one-subject-out cross-validation (LOSOCV) method, which is most appropriate as it accounts for between-participant variability [42]. The LOSOCV method sequentially trained the model on the data from all participants except for one, which was left out and used for testing. This procedure looped through the total number of participants, resulting in 17 kinematic prediction models. The dataset was separated into training, validation and test datasets. For each validation fold, data from 16 participants were separated into training (90%) and validation (10%) sets randomly based on the unique samples. Training data were used to optimise the model parameters, whereas validation data were used as the unseen data during the model training process to fine tune the parameters such as validation loss, batch size and learning rate. Finally, the model was tested on all samples for the left-out participant’s data. 

For each activity, we calculated the average root mean square error (RMSE), normalised RMSE (nRMSE) [43], mean absolute error (MAE) and Pearson correlation coefficient (R) between the Vicon reference and predictions for time-series data. R values were averaged across participants using Fisher’s z transformation [44]. The strength of the correlation was categorised as excellent (R > 0.9), strong (0.67 < R ≤ 0.9), moderate (0.35 < R ≤ 0.67) and weak (R ≤ 0.35) based on similar studies [29]. In addition, we calculated the RMSE for the average maximum (peakRMSE) and minimum (minRMSE) knee flexion angles. 

Each input variable and target variable were standardised for scale and distribution separately in each loop. Mean and standard deviation were computed only on the training data across all trials and all time points for each variable to prevent data leakage when applying pre-processing statistics. Validation and test data were standardised based on the corresponding mean and calculated from the training data used in each loop. A three-dimensional input shape is required for the BiLSTM model (N_samples, N_timesteps, N_features); therefore, input data are reshaped prior to being passed to the model. The number of samples collected from participants used to train the models is shown in Table 1. 

## 3. Results

**Figure 3:**
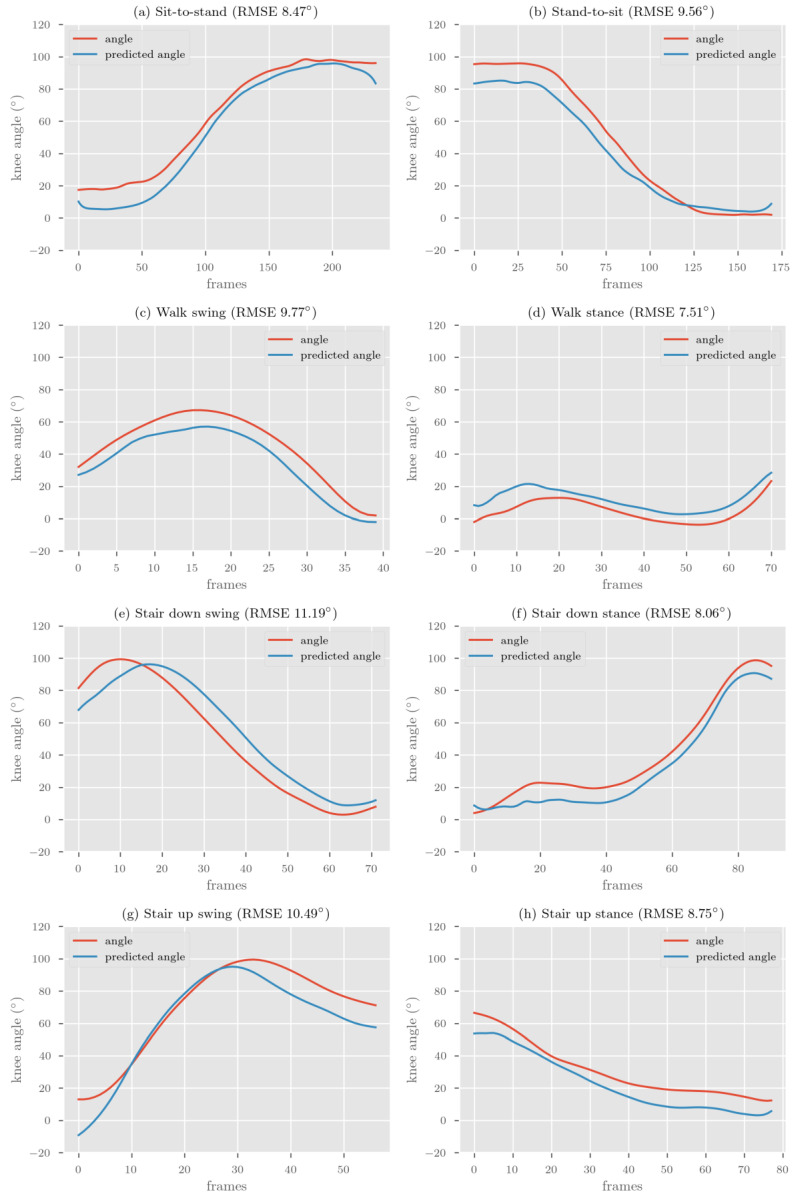
Representative single-leg BiLSTM model prediction compared to Vicon reference for each activity phase.

The participant characteristics are shown in Table 2. The accuracy of our two models is presented in Table 3. Examples of representative model prediction for each activity phase compared to the Vicon reference standard (based on RMSE) are presented in Figure 3. Overall, the difference between the double-leg and the single-leg model was small, with an RMSE difference ranging from 0.11° to 1.96° and MAE from 0.01° to 1.46° for time-series predictions. 

The single-leg model demonstrated the smallest RMSE/MAE across activities (five of eight activities) for time-series predictions compared to the double-leg model. The double-leg model demonstrated the smallest RMSE/MAE for predicting simultaneous double-leg activities (sit-to-stand and stand-to-sit) compared with activities that require reciprocal movements of both legs (walking or stairs). 

Correlations between the reference Vicon system and the deep learning model were excellent (R > 0.9) for both the single-leg and double-leg models for all activities except for the stance phase of walking. The strongest correlation coefficient was for sit-to-stand, stand-to-sit and the swing phase of ascending stairs (R = 0.99).

The peakRMSE and minRMSE for each activity was almost always lower than the time-series RMSE for large range activities (sit-to-stand, stand-to-sit and swing phases). For small range activities (stance phases), the minRMSE was always lower than the time-series RMSE, whereas the peakRMSE was always higher. 

## 4. Discussion

The aim of this study was to establish a proof-of concept for the feasibility of using IMU data collected from people who have knee osteoarthritis for development of a deep learning model to predict sagittal plane knee joint angles for multiple clinically relevant activities. We developed a BiLSTM kinematic prediction model on IMU training data that included walking, negotiating stairs and transitioning to/from a chair for people who have knee osteoarthritis. The prediction error (RMSE/MAE) between the reference Vicon system and kinematic predictions was lowest for the stance phase for walking and going down stairs, and highest for the swing phase for going down and up stairs. Although, as a proportion of the range used during each activity phase, the sit-to-stand and stand-to-sit had the lowest prediction error, the stance phase for walking and going down stairs had the highest prediction error (nRMSE). For time-series data, the shape of the predicted curve had a consistently excellent correlation (R > 0.9) to the Vicon system across activities. 

The second aim was to develop two types of models using training data from (i) two IMUs on one leg (single-leg) and (ii) four IMUs on two legs (double-leg). The single-leg model demonstrated more frequent smaller errors and had excellent correlations (R > 0.9) than the double-leg model across activities that require reciprocal, asymmetrical lower limb movement, such as walking and negotiating stairs; however, the difference in error between models was small. For activities that require bilateral simultaneous movement (sit-to-stand/stand-to-sit), the double leg model demonstrated smaller error. 

### 4.1. Comparison to Previous Literature

In comparison to other deep learning models were that developed to predict knee joint kinematics just for walking, our BiLSTM model for multiple activities demonstrated prediction errors and correlations within the range of previous studies (RMSE 0.97–12.1°, R 0.94–0.99) [22,24,25,26,27].

Only one other study has used deep learning to predict knee kinematics in people who have knee osteoarthritis for the activity of walking [26]. Our model has the benefit of being more broadly applicable for real world use, when combined with human activity recognition, because of the inclusion of multiple clinically important activities for people who have knee osteoarthritis. The model by Renani et al. [26] demonstrated small average RMSE 2.9° (SD 1.1) for time-series prediction of knee flexion/extension during walking using a BiLSTM model. Training data in that study was from four IMUs placed on the pelvis, thigh, shank and foot. In comparison, our BiLSTM model trained on data from only a thigh and shank IMU demonstrated substantially higher average RMSE during walking phases (single model—stance 7.04° (SD 2.6), swing 9.7° (SD 3.86)). Their results may have demonstrated lower error because of the higher number of samples (n = 3943) compared to our study (n = 955), the additional IMUs placed on the pelvis and foot, the inclusion in our training data of activities other than walking, or the difference in validation approach. 

For validation of a IMU prediction model to be meaningful to a clinician, it has been suggested that the average level of error for each new person should be reported [42], which is a strength of the LOSOCV method compared to the other validation methods (e.g., k-fold cross validation). Our results are similar to those of previous studies that use LOSOCV, rather than studies that use other validation approaches (e.g., [22,25,26,27]), and studies that use real IMU data compared to those that use simulated IMU data (see Section 4.3.3. Augmented and Simulated Data). For example, Wouda et al. [24] validated a LSTM model using IMU training data collected from a healthy population for time-series prediction of knee flexion during running. They used LOSOCV and reported an average RMSE of 12.1° (SD 1.5). In comparison, our model achieved lower average RMSE for walking swing and walk stance of 9.7° (SD 3.8) and 7.0° (SD 2.6). Although our model demonstrated lower average error, there was higher variability, which may be the result of using training data that included multiple activities rather than the single activity of walking. Our model also demonstrated good ability to predict the shape of the kinematic curve with excellent correlations (R > 0.9) for time-series prediction of all but one activity, comparing well to the models by Wouda et al. [24] (R = 0.94) and Renani et al. [26] (R = 0.99). 

Using raw accelerometer and gyroscope data for training deep learning prediction models appears a promising tool to aid clinical decision making for clinicians managing people with movement disorders, such as knee osteoarthritis, as it mitigates the requirement for the magnetometer, which is prone to interference, especially in free-living conditions where the magnetic field is not uniform [45]. However, deep learning approaches using real IMU data for the prediction of knee kinematics have not yet reached the consistent low error achieved by Kalman filter-based approaches that report RMSE as low as 1° for multiple clinically relevant activities [19] or 5.04° using the proprietary software for the IMUs described in this study [14]. Various clinical, data handling and machine learning architecture considerations may help to reduce prediction error in future studies.

### 4.2. Clinical Considerations for Kinematic Prediction Models

Development of various machine learning models has the potential to have a significant impact for clinical populations, such as for people who have knee osteoarthritis. However, the majority of these studies have not described the clinical implications of such models; therefore, this section discusses clinical considerations for future development of machine and deep learning models for prediction of joint kinematics.

#### 4.2.1. Variability of Movement in Clinical Populations

Although our model demonstrated a very small error and a high correlation for some participants, this was not the case across all participants. It is well established that people who have musculoskeletal or neurological health conditions move differently than healthy populations [8,46,47]. Across people who have knee osteoarthritis, there is diversity in movement patterns during functional activities related to disease severity [46]. Because of this heterogeneity of movement patterns across different conditions and even within a single diagnosis such as like knee osteoarthritis, it is important that models are trained and tested on the intended population for use. For example, Renani et al. [48] trained a CNN to predict spatiotemporal kinematics of the lower limb for people with knee osteoarthritis and after total knee replacement. Their model demonstrated consistently higher prediction error and variability across 12 spatiotemporal gait parameters for people that have knee osteoarthritis compared to people who had total knee replacement. Other studies have reported that the accuracy of human activity recognition models derived on data from healthy populations has had substantially reduced test accuracy in people who have health conditions, such as Parkinsonism [49,50]. It is currently unknown if the test accuracy of kinematic prediction models differs across populations. Given that people with knee osteoarthritis move differently and more variably than healthy people, the ability to generalise the kinematic prediction model accuracy between those populations should not be assumed. Future studies should consider testing prediction models on participants with health conditions of interest who demonstrate a range of movement impairments, and pain and disability levels. 

#### 4.2.2. Selecting Clinically Important Activities and Biomechanical Parameters

The clinically relevant use for predicting sagittal plane knee joint angles is to monitor biomechanics during functional activities in free-living environments and in-clinic to aid clinical decision making. Specifically, particular phases of activities (e.g., stance phase of ascending stairs) are of interest to clinicians because they are targets for rehabilitation. 

Prior to this study, machine learning models for predicting knee joint kinematics that could potentially be useful for people with knee osteoarthritis have only been trained and tested on walking data [21,22,23,24,25,26,27,48]. Unlike those studies where a kinematic prediction model was developed for walking, our model is the first to be trained and tested on a range of clinically relevant activities for a specific clinical population. Although walking is the most frequently performed activity of the lower limbs, we selected three activities (walking, negotiating stairs and transitioning to/from a chair) that are recommended as part of a clinical physical assessment in medical guidelines for knee osteoarthritis [9]. To improve clinical utility of machine learning prediction using IMU data, future studies should investigate kinematic prediction models for a broader range of clinically important activities. 

There are a broad range of kinematic and kinetic movement parameters that are of interest to clinicians and researchers for people with knee osteoarthritis [6]. Therefore, monitoring sagittal plane knee joint angles is only one movement parameter that could be recorded for clinically relevant activities in free-living conditions. Other movement parameters are also of interest because of their relationship with structural progression of knee osteoarthritis. Knee adduction moment, for example, is associated with the progression of medial compartment knee osteoarthritis [51,52]. There is early work investigating spatiotemporal kinematics [48], predicting knee moments and forces using deep learning approaches such as LSTM, CNN and ANN for the purposes of field monitoring [27,29,30]. To improve clinical utility of IMU machine/deep learning prediction models using IMU data, future studies should investigate integrating [12] human activity recognition [28] with both kinematic and kinetic prediction models (see Section 4.3.1) for a broad range of clinically relevant activities that include but are not limited to the activity of walking. 

#### 4.2.3. Reducing the Burden for Clinicians

Some studies use up to 17 IMUs across the whole body to train deep learning models for kinematic prediction of the lower limbs [24,48]. It is generally thought that having additional IMUs results in improved accuracy and reduced error for machine learning predictions using IMU data for human activity recognition [53,54]. However, having to use additional IMUs can be burdensome for clinicians. Our findings are similar to Hendry et al. [40], who investigated kinematic prediction for the hip and lumbar spine using IMUs to train a deep learning model for ballet dancers. They reported that their kinematic prediction model trained on only two IMUs placed on the lower limbs demonstrated less error (7.0°) than models that included additional training data from IMUs placed on the spine (7.8°). A novel finding in our study was that the prediction error with using data from only two IMUs on a single leg was less than that using four IMUs on two legs, which may be because of the asymmetrical and diverse nature of movement patterns that exist in people with knee osteoarthritis. Future studies should aim to determine the minimum number of IMUs required for specific conditions and activities, to reduce clinician burden. 

### 4.3. Considerations for Future Data Handling and Machine Learning Models

#### 4.3.1. Developing Data Handling Pipelines

Previously published kinematic [21,22,23,24,25,26,27,48] and kinetic [27,29,30] prediction models are currently only useful in conditions in which the wearer of the IMUs is observed, such as in a clinical environment. This is because in free-living conditions people wearing IMUs will perform other activities in addition to walking (e.g., transitioning to/from a chair and negotiating stairs). Therefore, biomechanical prediction models have limited use in free-living conditions without additional data processing that can automate the identification and labelling the long, continuous streams of data that are produced by IMUs.

Our approach was to train the kinematic prediction model on labelled data that could potentially be output from a human activity recognition deep learning algorithm as part of a data handling pipeline. We previously established a proof-of-concept about the development of a human activity recognition model [28] that can segment data into the phases of clinically important activities described in this study, which could be the first component of a data-handling pipeline.

However, it is currently unknown which method of data segmentation is most useful, minimally burdensome for clinicians and computationally efficient. We selected phases of activities because clinicians are typically interested in data from phases, rather than the whole gait cycle (see Section 4.2.2.). Data in other studies has been segmented in a variety of ways including continuous walking [22], three gait cycles [21], or single gait cycles [26,27]. The higher-order data segmentation in those studies may prove to be clinically useful for use in a human activity recognition model that includes other activities (e.g., going up stairs or sit-to-stand), and integration with a gait event detection algorithm [55].

#### 4.3.2. Single vs. Multiple Models

We developed a single kinematic prediction model to include training data from multiple activities, which provides more generalisability and precludes the need to model every activity [30]. However, there is uncertainty about the superiority of universal single models for prediction of kinematics across multiple activities compared to multiple models that predict only specific activities. Stetter et al. [30] reported the development of an ANN to predict knee joint forces for 16 sports specific activities (e.g., walking, running, jumping, and cutting). They noted the possibility that their model had higher error compared to the study by Wouda et al. [24] was because of their use of a single model for the multiple activities [30]. Contrary to this, our single model for multiple activities had lower RMSE [24] and stronger correlations [21] than other approaches that only used training data from a single activity. 

Furthermore, our double-leg model performed better than the single-leg model for bilateral simultaneous activities of sit-to-stand and stand-to-sit. Stetter et al. [30] demonstrated a similar effect where there a single-leg model had higher error for two leg activities (jump take-off and two leg jump landing) compared to single-leg activities. 

These results together may indicate that, in future studies, activity-specific models should be directly compared to models trained to predict kinematics for multiple activities, and that models trained on both legs may impact the results of asynchronous movement such as walking. 

#### 4.3.3. Augmented and Simulated Data

One challenge of developing generalisable kinematic prediction models is the collection of a sufficient number of samples from a representative cohort of participants, a process which is burdensome. This challenge is highlighted by the large RMSE/MAE for the stair down stance, where there was the least number of training data for the model. One solution becoming increasing popular is to augment the training data by including simulated IMU data [23,26]. It has been demonstrated that adding simulated data results in a 27–45% improvement in RMSE, resulting in knee flexion RMSE between 1.4–5.22° [26,56]. These approaches using simulated and augmented data may provide additional benefit in models trained on data collected from clinical populations, such as people with knee osteoarthritis. In addition, using augmented data may help reduce the impact of misplacement of sensors by either clinicians or patients.

#### 4.3.4. Deep Learning Architecture

We used BiLSTM following on from the work of Renani et al. [26]. BiLSTM is proposed to improve prediction accuracy because it transverses the input data twice, using both past and future data points, compared to traditional LSTM [39]. Future studies should investigate the performance of BiLSTM compared to traditional LSTM and other deep learning approaches for kinematic prediction. Although we used BiLSTM, there is some indication that combining multiple deep learning architectures, such as CNN with LSTM (ConvLSTM), can improve prediction accuracy for IMU data [57]. Hernandez et al. [22] demonstrated that this combined deep learning approach using ConvLSTM can provide good results for knee flexion time-series predictions (MAE 3 (SD 1.15), R = 0.99) using a nested k-fold validation using a 70% training, 15% validation and 15% test approach [22]. Researchers must further investigate the balance between predictive accuracy and the requirement for pre-processing of data. Mundt et al. [38] tested the predictive accuracy of a CNN, LSTM and multilayer perceptron network for lower limb kinematics and kinetics. They demonstrated superior accuracy with a CNN for prediction of kinematics, although the pre-processing requirements are high for this type of model compared to LSTM, which may be more suited to real-time applications. 

## 5. Limitations

Because this study was a proof-of-concept investigation, there are a number of limitations. We included only 17 participants, did not have a representative number of female participants, and excluded people with high BMI. These factors may limit the generalisability of our model for the broader population with knee osteoarthritis. Further, there was an unbalanced dataset with a significantly different number of trials across activities, which may have affected the results. This study included clinically relevant activities described in clinical guidelines for people who have knee osteoarthritis [9]. However, there are additional activities that people perform daily that were not included. A single model for predicting kinematics for multiple activities was used in this study, which may have affected the prediction error, and it is unclear if a universal model is feasible for all activities a person may perform. Further investigation is required to determine the comparative accuracy of a single model for multiple activities versus individual models for each type of clinically relevant activity (or phase of activities) in clinical populations, such as people who have knee osteoarthritis.

## 6. Conclusions

This proof-of-concept study demonstrates that using IMU training data collected from people who have knee osteoarthritis to predict sagittal plane knee joint kinematics during multiple clinically important activities using a deep learning model is feasible. Our novel BiLSTM model demonstrated that using training data from as few as two IMUs placed on one leg performs with less error for most activities than with additional training data from IMUs on both legs. To be of clinical value, the model presented in this study could be combined with a human activity recognition system to monitor response to treatment in people with knee osteoarthritis. 

## Figures and Tables

**Table 1:** Number of samples for each activity.

Phase of Activity	Samples (Participants)
Sit-to-stand	61 (15)
Stand-to-sit	61 (15)
Walk swing	245 (17)
Walk stance	244 (17)
Stair up swing	130 (15)
Stair up stance	87 (15)
Stair down swing	83 (15)
Stair down stance	44 (15)
Total	955 (17)

**Table 2:** Characteristics of participants.

	All Participants (n = 17)	
Characteristics	Mean	SD
Age (years)	66.2	8.7
Male (%)	59%	
Weight (kg)	80.3	15.9
Height (cm)	173	8.8
BMI (kg/m^2^)	26.6	15.9
KOOS function	68.4	12.6

BMI = body mass index, cm = centimetres, kg = kilograms, KOOS = Knee injury and Osteoarthritis Outcome Scale, m = metres, SD = standard deviation.

**Table 3:** Knee flexion angle prediction error for time-series, peak and minimum estimates for each activity.

Single-Leg Prediction Model									
				Walk		Stair Down		Stair Up	
Outcome		Sit- to-stand	Stand- to-sit	Swing	Stance	Swing	Stance	Swing	Stance
Time- Series	RMSE (°) (SD)	8.24	9.30	9.70	7.04	11.78	8.22	10.41	8.99
		(3.02)	(2.99)	(3.86)	(2.60)	(6.04)	(2.80)	(5.11)	(3.70)
	nRMSE (%) (SD)	9.79	10.86	17.66	36.33	14.06	22.91	15.06	19.14
		(3.71)	(3.78)	(9.05)	(14.39)	(7.90)	(9.99)	(8.70)	(10.00)
	MAE (°) (SD)	7.12	7.96	8.46	5.99	10.37	7.00	9.06	8.06
		(2.87)	(2.60)	(3.45)	(2.34)	(5.44)	(2.55)	(4.54)	(3.64)
	R	0.99	0.99	0.98	0.85	0.99	0.96	0.98	0.98
Peak	RMSE (°) (SD)	6.46	6.89	9.75	10.31	9.72	21.38	9.78	11.73
		(2.48)	(4.28)	(6.21)	(5.42)	(3.72)	(12.29)	(6.65)	(6.39)
Minimum	RMSE (°) (SD)	6.92	7.71	7.35	6.21	8.07	6.07	10.33	8.04
		(4.57)	(5.77)	(3.72)	(2.99)	(5.73)	(4.69)	(5.00)	(5.76)
**Double-Leg Prediction Model**									
				Walk		Stair Down		Stair Up	
Outcome		Sit- to-stand	Stand- to-sit	Swing	Stance	Swing	Stance	Swing	Stance
Time- Series	RMSE (°) (SD)	7.27	8.10	9.81	8.19	12.85	10.19	10.17	9.61
		(1.72)	(2.29)	(3.98)	(2.69)	(5.63)	(3.19)	(4.63)	(3.59)
	nRMSE (%)(SD)	8.68	9.45	17.78	43.33	15.70	32.93	15.14	19.90
		(2.58)	(2.89)	(8.68)	(16.55)	(7.45)	(23.18)	(8.29)	(8.50)
	MAE (°) (SD)	6.03	6.72	8.47	6.92	11.09	8.47	8.81	8.36
		(1.69)	(2.11)	(3.52)	(2.39)	(5.07)	(3.02)	(4.25)	(3.40)
	R	0.99	0.99	0.97	0.74	0.98	0.92	0.98	0.96
Peak	RMSE (°) (SD)	5.09	6.44	9.23	10.29	10.73	24.33	10.01	13.28
		(2.97)	(4.23)	(5.65)	(6.51)	(5.39)	(10.70)	(8.22)	(8.18)
Minimum	RMSE (°) (SD)	6.49	6.15	8.76	6.60	11.21	8.99	10.36	7.79
		(4.55)	(4.13)	(4.31)	(2.37)	(8.60)	(3.79)	(5.02)	(5.36)

° = degrees of movement, MAE = mean absolute error, R = Pearson correlation coefficient, RMSE = root mean squared error, nRMSE = normalised RMSE, SD = standard deviation.

## Data Availability

The data presented in this study may be available on request from the corresponding author.
